# Medical Image-Based Hemodynamic Analyses in a Study of the Pulmonary Artery in Children With Pulmonary Hypertension Related to Congenital Heart Disease

**DOI:** 10.3389/fped.2020.521936

**Published:** 2020-12-02

**Authors:** Liping Wang, Jinlong Liu, Yumin Zhong, Mingjie Zhang, Jiwen Xiong, Juanya Shen, Zhirong Tong, Zhuoming Xu

**Affiliations:** ^1^Department of Thoracic and Cardiovascular Surgery, Shanghai Children's Medical Center, Shanghai Jiao Tong University School of Medicine, Shanghai, China; ^2^Pediatric Translational Medicine Institute, Shanghai Jiao Tong University School of Medicine, Shanghai, China; ^3^Shanghai Engineering Research Center of Virtual Reality of Structural Heart Disease, Shanghai Children's Medical Center, Shanghai Jiao Tong University School of Medicine, Shanghai, China; ^4^Department of Radiology, Shanghai Children's Medical Center, Shanghai Jiao Tong University School of Medicine, Shanghai, China

**Keywords:** pulmonary hypertension, energy loss, congenital heart disease, computational fluid dynamics, wall shear stress

## Abstract

**Objective:** Pulmonary hypertension related to congenital heart disease (PH-CHD) is a devastating disease caused by hemodynamic disorders. Previous hemodynamic research in PH-CHD mainly focused on wall shear stress (WSS). However, energy loss (EL) is a vital parameter in evaluation of hemodynamic status. We investigated if EL of the pulmonary artery (PA) is a potential biomechanical marker for comprehensive assessment of PH-CHD.

**Materials and Methods:** Ten PH-CHD patients and 10 age-matched controls were enrolled. Subject-specific 3-D PA models were reconstructed based on computed tomography. Transient flow, WSS, and EL in the PA were calculated using non-invasive computational fluid dynamics. The relationship between body surface area (BSA)-normalized EL ($\overset{.}{E}$) and PA morphology and PA flow were analyzed.

**Results:** Morphologic analysis indicated that the BSA-normalized main PA (MPA) diameter (D_MPAnorm_), MPA/aorta diameter ratio (D_MPA_/D_AO_), and MPA/(left PA + right PA) [D_MPA_/D_(LPA+RPA)_] diameter ratio were significantly larger in PH-CHD patients. Hemodynamic results showed that the velocity of the PA branches was higher in PH-CHD patients, in whom PA flow rate usually increased. WSS in the MPA was lower and $\overset{.}{E}$ was higher in PH-CHD patients. $\overset{.}{E}$ was positively correlated with D_MPAnorm_, D_MPA_/D_AO_, and D_MPA_/D_(LPA+RPA)_ ratios and the flow rate in the PA. $\overset{.}{E}$ was a sensitive index for the diagnosis of PH-CHD.

**Conclusion:** $\overset{.}{E}$ is a potential biomechanical marker for PH-CHD assessment. This hemodynamic parameter may lead to new directions for revealing the potential pathophysiologic mechanism of PH-CHD.

## Introduction

Congenital heart disease (CHD) is an anatomic defect associated with abnormal cardiovascular development *in utero*. Medical research shows that CHD prevalence at birth has increased from 0.6 to 0.9% in recent years, and that Asia has the highest prevalence of all regions evaluated (1).

In recent years, although advances in cardiac surgical skills and perioperative management have reduced CHD-related mortality dramatically, CHD remains the leading cause of death due to birth defects in the first year of life (2). The mortality increases if it is accompanied by severe complications.

Pulmonary hypertension (PH) is a common complication of CHD that can start at any age. Timely and accurate diagnosis reduces the severity of pulmonary vascular remodeling and the risk of heart failure, thereby giving patients a chance to receive the best course of treatment and long-term outcomes.

However, the diagnosis and prognosis of PH have not improved much in recent years (3). The main reason is a lack of understanding of its pathophysiology. PH is a response to abnormal hemodynamics in CHD patients (4–6), which appear before morphologic remodeling (7). Therefore, knowing the hemodynamic characteristics of the pulmonary artery (PA) may benefit comprehension of pathophysiologic mechanisms and yield more detailed information for the diagnosis and treatment of PH in CHD (PH-CHD).

Right-heart catheterization and transthoracic echocardiography (TTE) are used commonly for PH-CHD evaluation. However, neither the invasive catheterization nor the non-invasive TTE can be used to provide detailed information on hemodynamics.

Thanks to the development of computer and medical imaging technologies, computational fluid dynamics (CFD) has been employed to obtain local hemodynamics of the measured site and display them in a visual and stereoscopic way, which enables the study of the relationship between hemodynamics and CHD development. In recent years, CFD has been used to illustrate hemodynamic characteristics in patients with PH (8, 9). Many of those studies have concentrated mainly on assessment of shear stress (9, 10). Nevertheless, it has been shown that increased energy loss (EL) is closely related to the long-term outcome of patients (11–13).

EL is closely related to vascular morphology and flow patterns, and it is a crucial parameter in evaluation of hemodynamic disorders. Lee and colleagues (14) studied EL in the PA in different pathophysiologic scenarios and concluded that EL increased in patients with abnormal pulmonary vascular morphology. A recent study in adult patients with chronic thromboembolic pulmonary hypertension (CTEPH) demonstrated that alteration of energy dissipation in the PA has substantial effects on disease development (15).

In children with PH-CHD, abnormal morphology of the PA and flow status may influence EL. Few studies have investigated this biomechanical factor and its potential effect on this population.

We used CFD to explore subject-specific hemodynamics, including wall shear stress (WSS) and EL, which are expected to assist better understanding of PH-CHD pathophysiology and clinical decision making. For controlling intergroup variation caused by age, body surface area (BSA) was used to normalize quantitative indices.

## Materials and Methods

### Ethical Approval of the Study Protocol

The study protocol was approved by the Health Research Ethics Board of Shanghai Children's Medical Center within Shanghai Jiao Tong University School of Medicine (Shanghai, China). Written informed consent was obtained from the parent/legal guardian of participants.

### Patient Selection

We enrolled 10 PH-CHD patients with a velocity of tricuspid regurgitation (TR) ≥4.0 m/s and/or a predominantly right-to-left shunt of the ventricular septum, which was measured by TTE. Meanwhile, a control group of 10 age-matched CHD patients without PH were recruited to exclude confounders and control intergroup variation for better single-factor analysis. The diagnosis of the control group was established when TTE showed a velocity of TR ≤ 2.9 m/s and/or left-to-right shunt of the ventricular septum ≥4.0 m/s. The enrollment criteria followed the guideline of PH, which was the general consent achieved at the 6th World Symposium on Pulmonary Hypertension (16, 17). Detailed clinical information on these patients is shown in Table 1.

**Table 1:** Patient-specific clinical data.

	**Patients**	**Diagnosis**	**Shunt size (cm)**	**Shunt velocity (m/s)**	**TR (m/s)**	**PI (m/s)**
Non-PH	1	VSD	0.75	4.00 (left to right)	Mild	Mild
	2	CoA	/	/	Mild	2.06
	3	VSD	0.66	4.19 (left to right)	Mild	Mild
	4	VSD	0.70	4.80 (left to right)	Mild	Mild
	5	VSD	0.87	4.76 (left to right)	Mild	Mild
	6	VSD/ASD	1.05 (VSD)	4.95 (left to right, VSD)	Mild	Mild
	7	VSD	1.25	4.03 (left to right)	Mild	Mild
	8	VSD/ASD	0.77 (VSD)	5.00 (left to right, VSD)	Mild	Mild
	9	PAPVC/ASD	0.66 (II ASD)	/	2.70	Mild
	10	PAPVC/ASD	1.52 (II ASD)	/	2.80	1.85
PH-CHD	1	Cor (obstructed)/PDA	0.1 (PDA)	2.09 (right to left)	5.00	Mild
	2	Supracardiac TAPVC (obstructed)/ASD	1.38	right to left	4.10	Mild
	3	VSD	1.1	Bi-directional	4.28	3.67
	4	VSD	0.89	Bi-directional	4.68	4.59
	5	VSD/ASD/PDA	0.97/0.15 (VSD/PDA)	Bi-directional	5.62	Mild
	6	CAVC/PDA	0.96/0.21 (VSD/PDA)	Bi-directional	/	Mild
	7	VSD/ASD	0.70 (VSD)	Bi-directional	4.66	Mild
	8	Supracardiac TAPVC (obstructed)/VSD/ASD	0.40 (VSD)	Bi-directional	4.79	4.04
	9	CAVC/PDA	2.00/0.28 (VSD/PDA)	Bi-directional	/	3.00
	10	VSD	1.93	Bi-directional	/	4.01

*VSD, ventricular septal defect; CoA, coarctation; ASD, atrial septal defect;PDA, patent ductus arteriosus;CAVC, atrioventricular septal defect;BSA, body surface area; PAPVC, partial anomalous pulmonary venous drainage; Cor, cor triatriatum; TAPVC, total anomalous pulmonary venous drainage; TR, tricuspid regurgitation; PI, pulmonary insufficiency*.

All the clinical data of patients were collected: sex, age, weight, height, BSA, body mass index (BMI), left ventricular ejection fraction (LVEF), and contrast-enhanced computed tomography (CT) of the chest.

We excluded individuals with stenosis of the right ventricular outflow tract, pulmonary disease, or other diseases that could be an underlying cause of PH.

### Model Reconstruction

**Figure 1:**
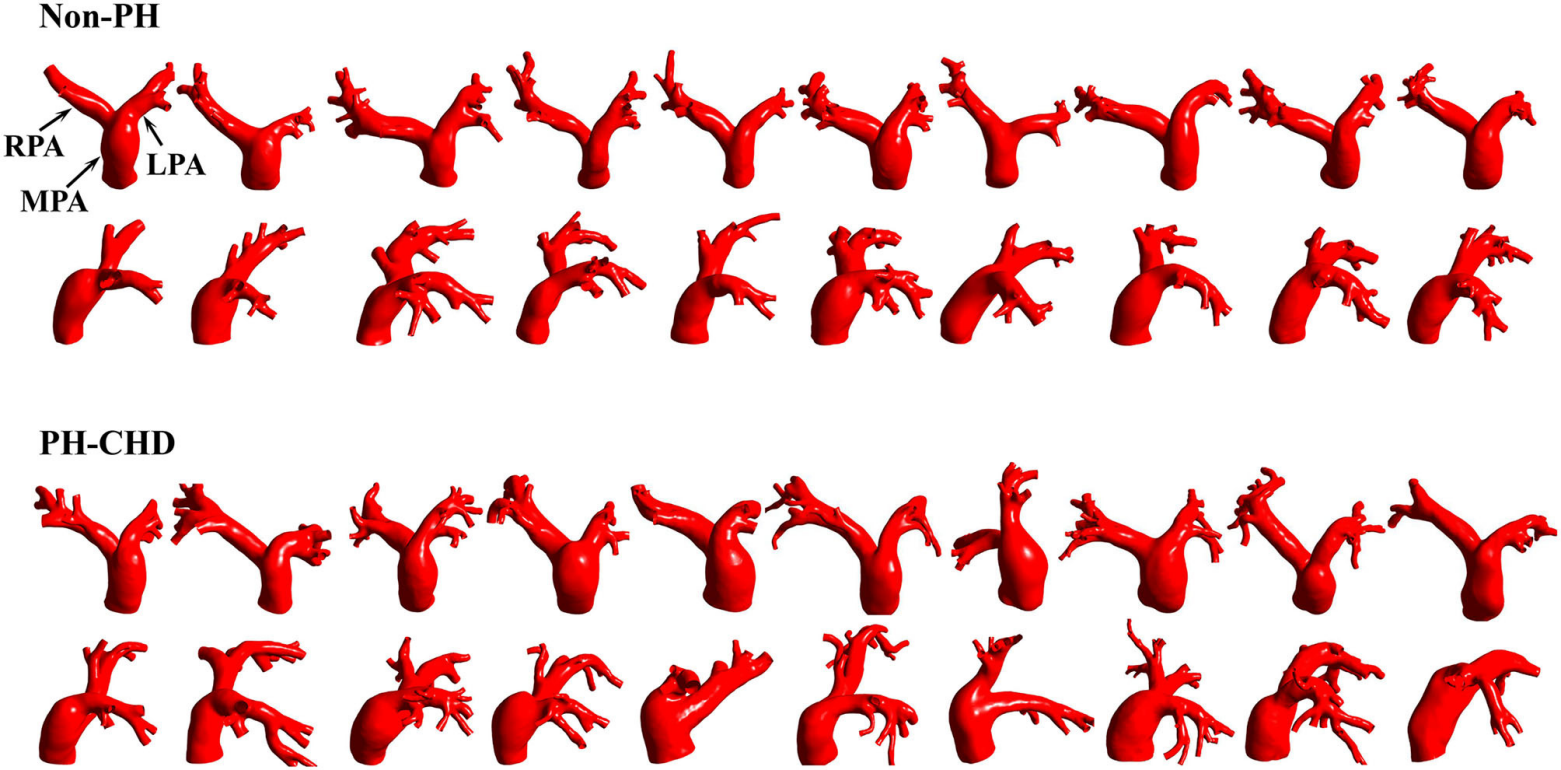
Subject-specific 3-D models of the proximal pulmonary artery (MPA, main pulmonary artery; LPA, left pulmonary artery; RPA, right pulmonary artery).

Sixty-four-row contrast-enhanced volumetric CT (Discovery CT750 HD; General Electric, Boston, MA, USA) data were used for reconstruction of 3-D subject-specific PA models in Mimics 20.0 (Materialize, Leuven, Belgium) and surface smoothing in 3-Matic 11.0 (Materialize, Leuven, Belgium). Figure 1 shows the lateral and anterior views of the 3-D-reconstructed PA geometries of these 20 patients.

The maximum diameter of the main PA (D_MPA_), left PA (D_LPA_), and right PA (D_RPA_) was measured. Subject-specific aorta models were reconstructed to obtain the maximum diameter of the aorta (D_AO_). These vascular parameters were normalized by BSA to control for age-related deviation. The D_MPA_/D_AO_ and D_MPA_/D_(LPA+RPA)_ ratios were calculated to compare morphologic differences among different vessel segments.

### Governing Equations

We assumed that the PA flow was that of an incompressible Newtonian fluid. The Navier–Stoke (N-S) equations (1) were used to describe the 3-D blood flow in the PA, which has a constant density (ρ = 1,060 kg/m^3^) and viscosity (μ = 4.0 × 10^−3^ Pa s).

(1) $$\begin{array}{l} \{\begin{array}{l} \frac{\partial }{\partial t}\left(\rho u_{i}\right)+\frac{\partial }{\partial x_{j}}\left(\rho u_{i}u_{j}\right)=\frac{\partial p}{\partial x_{i}}+\frac{\partial }{\partial x_{j}}\left[\mu \left(\frac{\partial u_{i}}{\partial x_{j}}+\frac{\partial u_{j}}{\partial x_{i}}\right)\right]+f_{i}\\ \frac{\partial \rho }{\partial t}+\frac{\partial }{\partial x_{j}}\left(\rho u_{j}\right)=0 \end{array} \end{array}$$

where *i, j* = 1, 2, 3; *x*_1_*, x*_2_*, x*_3_, represents the coordinate axes; *u*_*i*_*, u*_*j*_ are velocity vectors; *p* is pressure; *t* is time; and *f*_*i*_ indicates the action of body forces and was omitted in the practical calculation. The average Reynolds number among all models ranged from 2,700 to 5,500. The maximum Reynolds number ranged from 5,700 to 13,000. Thus, we assumed the flow in the PA was turbulent, and we used a standard *k*-ε model to solve complex pulsatile flow.

### Mesh Generation

Mesh generation was undertaken to discretize the computational domain and solve the governing equations using commercial software (ANSYS®-ICEM CFD 2019; Canonsburg, PA, USA). We used tetrahedral grids to discretize the volume layers of the fluid domain. Three body-fitted prism layers were used to improve the accuracy of calculation of the boundary layer of WSS. Grid independence was carried out to find the optimized mesh for CFD simulation, and the results were stable with a grid number reaching 0.9 million. The meshes of PH-CHD patients and controls were ~1.5 and ~1 million elements, respectively.

### Boundary Conditions and Calculation

**Figure 2:**
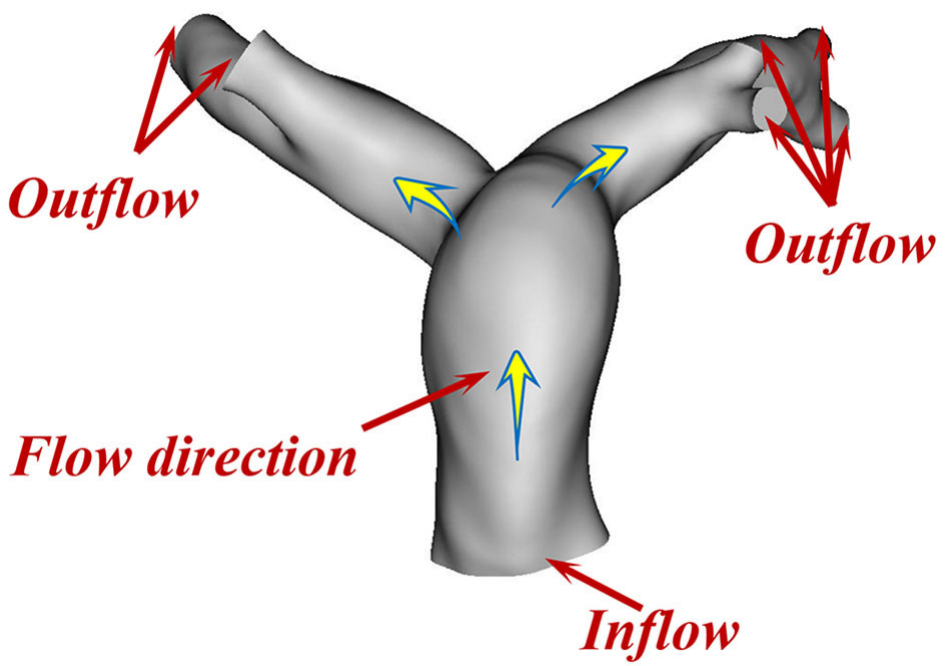
Inflow and outflow in the PA (schematic).

We used the pulsatile velocity of the MPA obtained by TTE as the inlet boundary condition. In addition, we assumed relative pressure as the outlet boundary condition and rigid with no-slip boundary conditions for the vessel wall. Figure 2 shows the inflow and outflow in the PA schematically.

We used ANAYS®-CFX 2019 (Canonsburg, PA, USA) to solve the blood flow in the PA. The convergence criteria were set to 10^−5^ for each time step. More detailed information of the method has been reported in our previous work (13, 18–20).

### Hemodynamic Evaluation

Transient PA streamlines, WSS, and EL were calculated to evaluate the biomechanical differences of patients with and without PH-CHD.

To better display the flow pattern during a cardiac period, we calculated the velocity and flow pattern at six time points (a–f) in a cardiac cycle.

WSS demonstrates the frictional force between blood flow and the vessel wall and was determined using Equation (2, 21, 22):

(2) $$\begin{array}{l} \tau \;wall=-\mu {\frac{\partial ux}{\partial n}|}_{n=0} \end{array}$$

where *u*_x_ is the velocity of the fluid near the vessel wall and *n* is the height above the vessel wall.

EL is the energy difference between the inlet and outlet of the calculated domain and was calculated by Equation (3):

(3) $$\begin{array}{l} EL=E_{inlet}-E_{outlet} \end{array}$$

(4) $$\begin{array}{l} =\underset{inlet}{\sum }\left(P_{i}+\frac{1}{2}\rho u_{i}^{2}\right)Q_{i}-\underset{outlet}{\sum }\left(P_{0}+\frac{1}{2}\rho u_{0}^{2}\right)Q_{0} \end{array}$$

where *P* is the static pressure, *Q* is the flow rate, and *i, j* are the inlet and outlet of PA, respectively. To control for age-related variation among patients, the BSA normalized EL ($\overset{.}{E}$) was determined using Equation (4):

(5) $$\begin{array}{l} \overset{.}{E}=\frac{EL}{S} \end{array}$$

where *S* represents the BSA.

### Statistical Analysis

Statistical analyses were carried out using SPSS 23.0 (IBM, Armonk, NY, USA). The Shapiro–Wilk test was used to check the normality of all parameters. Data with a normal distribution were analyzed using Student's *t*-test. Associations between variables were analyzed by Pearson's correlation coefficient (*r*) with two-tailed probability (*p*). A receiver operating characteristic (ROC) curve was used to measure the sensitivity and specificity of the calculated parameters. A threshold *p* < 0.05 was considered significant.

## Results

### Demographic and Morphologic Analyses

Table 2 shows the demographic and morphologic measurements obtained in the two groups. The baseline data of sex, age, weight, height, BSA, BMI, and LVEF were similar in the two groups. The morphologic parameters of normalized D_LPA_ (D_LPAnorm_), normalized D_RPA_ (D_RPAnorm_), and normalized D_AO_ (D_AOnorm_) were not significantly different between the groups. However, the normalized D_MPA_ (D_MPAnorm_), D_MPA_/D_AO_, and D_MPA_/D_(LPA+RPA)_ ratios were, in general, larger in PH-CHD patients. These data indicate that the MPA was dilated markedly and there was relative stenosis in the LPA and RPA in PH-CHD patients.

**Table 2:** Subject-specific clinical data for the two groups.

	**PH-CHD group**	**Control group**	***p*-value**
Sex (male/ female)	6/4	3/7	0.370
Age (month)	35.1 ± 31.239	40.20 ± 25.961	0.696
Height (cm)	84.40 ± 22.965	97.00 ± 24.585	0.252
Weight (kg)	11.05 ± 5.459	14.77 ± 6.441	0.180
BSA (m^2^)	0.50 ± 0.209	0.60 ±0.229	0.221
BMI (kg/m^2^)	14.76 ± 1.106	15.39 ± 2.678	0.501
LVEF (%)	69.75 ± 11.306	66.55 ± 2.448	0.403
Normalized D_MPA_ (cm)	4.93 ± 1.568	3.29 ± 0.913	0.010^*^
Normalized D_LPA_ (cm)	2.63 ± 0.871	1.99 ± 0.454	0.058
Normalized D_RPA_ (cm)	2.88 ± 0.993	2.15 ± 0.500	0.058
Normalized D_AO_ (cm)	3.33 ± 1.089	2.89 ± 0.870	0.336
D_MPA_/D_AO_	1.50 ± 0.235	1.17 ± 0.240	0.006^*^
D_MPA_/D_(LPA+RPA)_	0.91 ± 0.115	0.79 ± 0.084	0.019^*^

*BSA, body surface area; BMI, body mass index; LVEF, left ventricular ejection fraction; D, diameter; MPA, main pulmonary artery; AO, aorta; LPA, left pulmonary artery; RPA, right pulmonary artery*;

* *p < 0.05*.

### Streamlines

The subject-specific streamlines of the PA were computed to investigate differences in flow patterns. Statistical analyses revealed that the flow rate in the PA [in L/(min·m^2^)] was significantly higher at the normalized mean (V_meannorm_) and maximum (V_maxnorm_) velocity in PH-CHD patients than in controls (19.63 ± 6.223 vs. 11.94 ± 3.651, *p* = 0.004; 50.05 ± 13.882 vs. 29.98 ± 8.031, *p* = 0.001).

**Figure 3:**
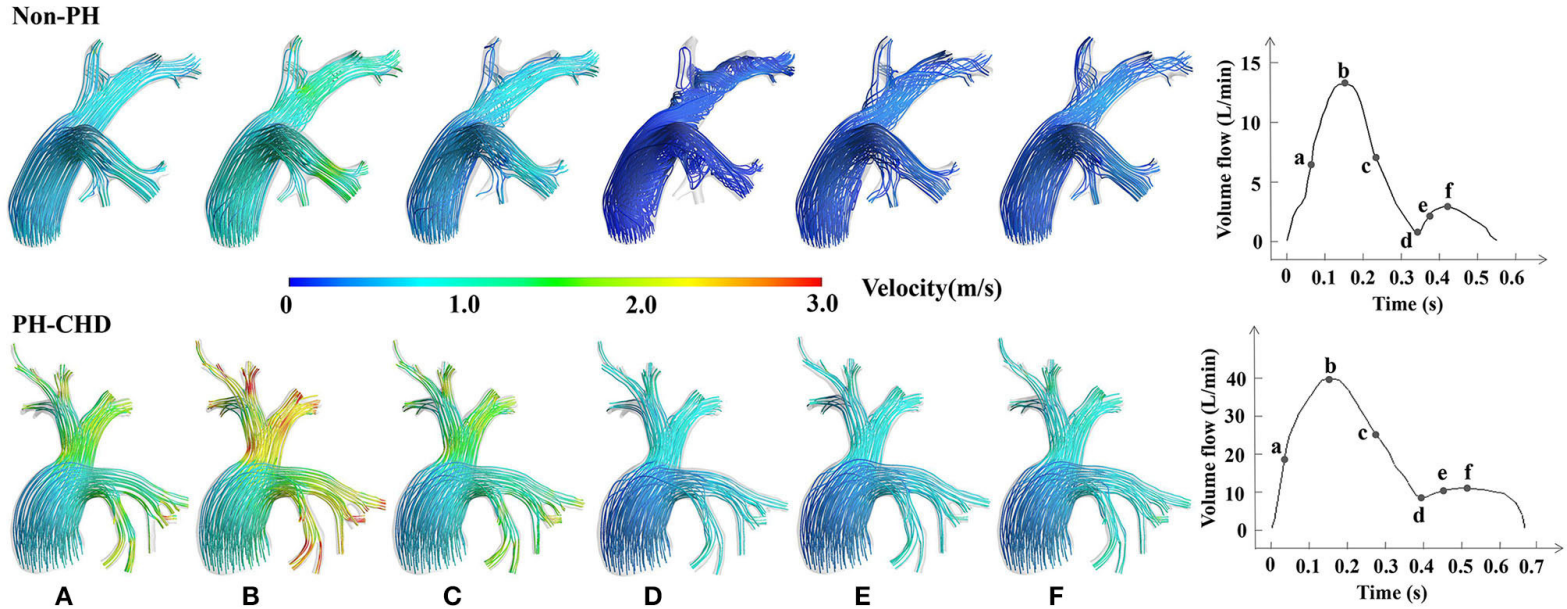
Subject-specific streamlines at six time points in one cardiac period. **(A)** The midpoint of the period from the beginning of the cardiac cycle to the highest velocity reached in the rapid ejection phase, which is assumed to show rapid variation in the velocity. **(B)** The time point at the highest velocity of blood flow in one cardiac cycle. **(C)** The midpoint of the period when the velocity of blood flow decreased from the highest to the lowest, which is assumed to show rapid variation in the velocity during the slow ejection phase. **(D)** The time point at the lowest velocity of blood flow in one cardiac cycle. **(E)** The midpoint of the period between point **d** and the time point at the highest velocity in diastole with rapid variation in the velocity observed in diastole. And **(F)**, The time at the highest velocity of diastole.

Figure 3 shows an example of the plots of PA flow rate in a cardiac cycle and the streamlines generated at six specific time points in two patients. One was a PH-CHD patient, and the other was a matched control subject. The velocity at the MPA was not significantly different between the two groups. However, at the PA branches, especially the bifurcation of the MPA into the LPA and RPA, the velocity was visibly higher in the PH-CHD patient than in the control subject. The velocity decreased faster in the non-PH patient than in the control subject during slow ejection and diastole (Figures 3C–F). Meanwhile, turbulent flow was observed at the same time point. Conversely, blood flow was relatively steady in the PH-CHD patient.

### Wall Shear Stress

**Figure 4:**
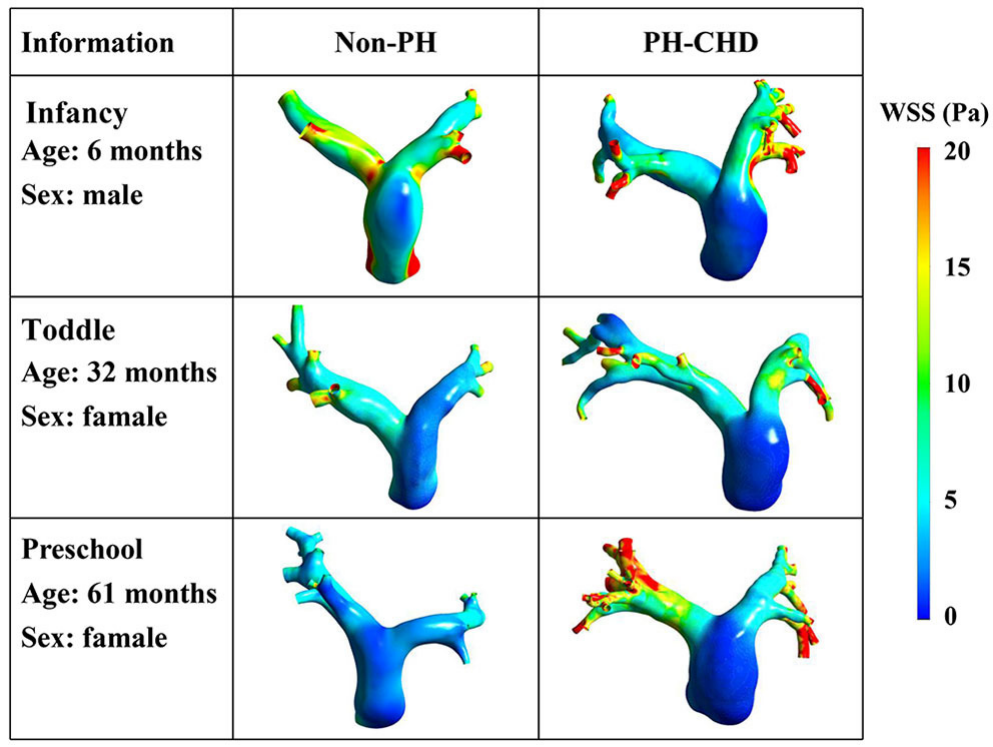
Distribution of wall shear stress at maximum velocity.

Figure 4 shows the results of detailed examination of the subject-specific WSS obtained in six patients of different ages. Simulated results demonstrated that the WSS of the MPA was visibly lower in PH-CHD patients than in control subjects. However, the spatially averaged WSS and time-averaged WSS were not significantly different between the two groups (7.35 ± 2.780 vs. 6.37 ± 3.978 Pa, *p* = 0.525; 2.41 ± 0.945 vs. 1.90 ± 0.983 Pa, *p* = 0.249). These average values could not reflect the local variation of WSS at the MPA, LPA, and RPA.

### Energy Loss

**Figure 5:**
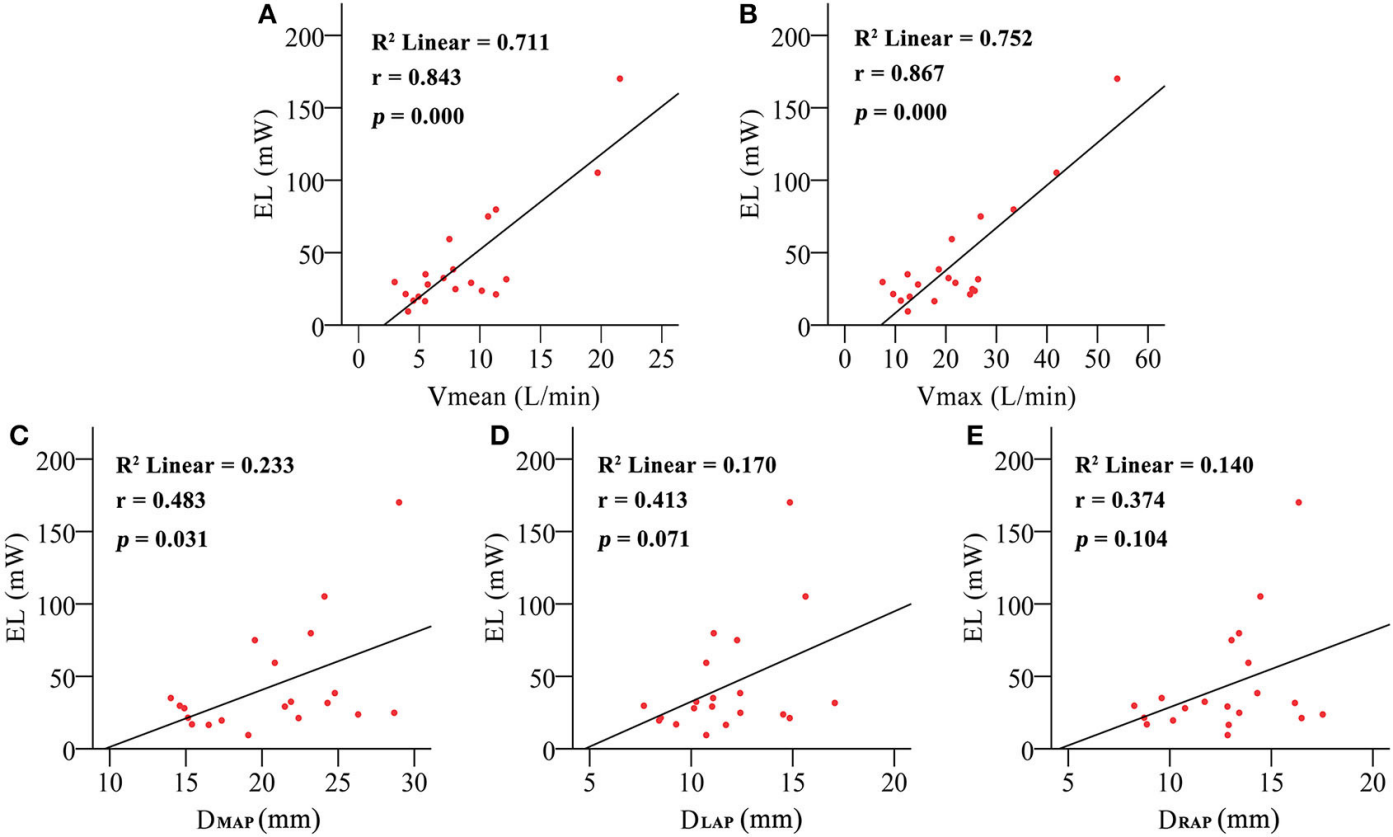
The correlation between energy loss (EL) and mean pulmonary artery inflow (V_mean_) **(A)**, maximum pulmonary artery inflow (V_max_) **(B)**, mean pulmonary artery diameter (D_MPA_) **(C)**, left pulmonary artery diameter (D_LPA_) **(D)**, and right pulmonary artery diameter (D_RPA_) **(E)**.

**Figure 6:**
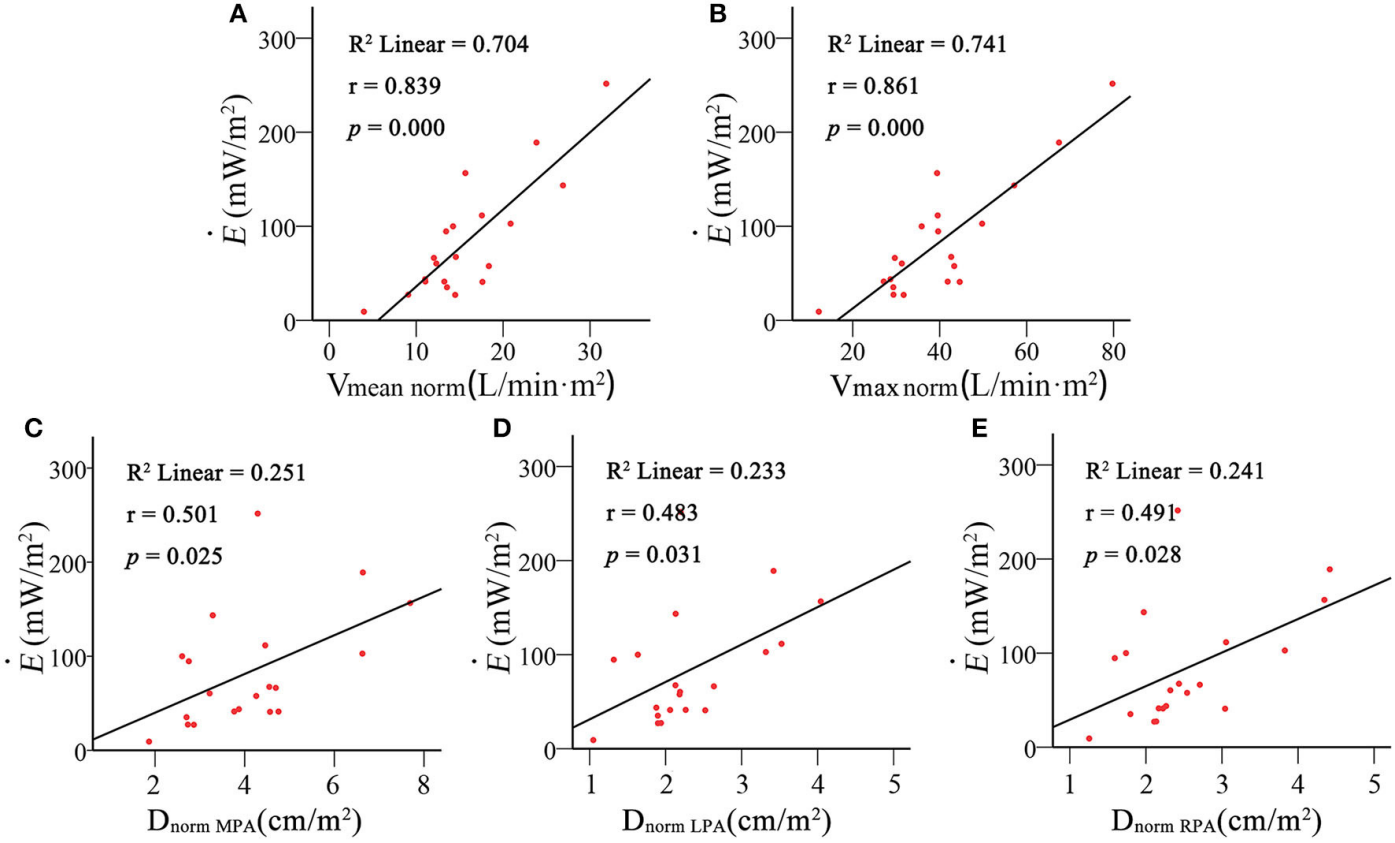
Correlation analysis of body surface area (BSA)-normalized energy loss ($\overset{.}{E}$) with BSA-normalized mean pulmonary artery inflow (V_meannorm_) **(A)**, maximum pulmonary artery inflow (V_maxmean_) **(B)**, mean pulmonary artery diameter (D_MPAnorm_) **(C)**, left pulmonary artery diameter (D_LPAnorm_) **(D)** and right pulmonary artery diameter (D_RPAnorm_) **(E)**.

EL of the PA was significantly higher in the PH group than in the control group (60.41 ± 46.551 vs. 26.34 ± 18.175 mW, *p* = 0.031). Figure 5 shows the relationship between EL and the flow rate and morphology of the PA. There's a positive correlation of EL and the flow rate of PA (Vmean: *r* = 0.843, *p* = 0.000; Vmax: *r* = 0.867, *p* = 0.000) but a weakened relationship with D_MAP_ (*r* = 0.483, *p* = 0.031) and no relationship with D_LPA_ (0.413, 0.071) and _DRPA_ (0.374, 0.104). $\overset{.}{E}$ was calculated to control the intergroup variation caused by age.$\overset{.}{E}$ was increased predominantly in the PH group (121.60 ± 64.820 vs. 45.15 ± 25.302 mW/m^2^, *p* = 0.007). Figure 6 shows the $\overset{.}{E}$ was positively correlated with D_MPAnorm_ (*r* = 0.501, *p* = 0.025), D_LPAnorm_ (0.483, 0.031), D_RPAnorm_ (0.491, 0.028), V_meannorm_ (0.861, 0.000), and V_maxnorm_ (0.839, 0.000).

**Figure 7:**
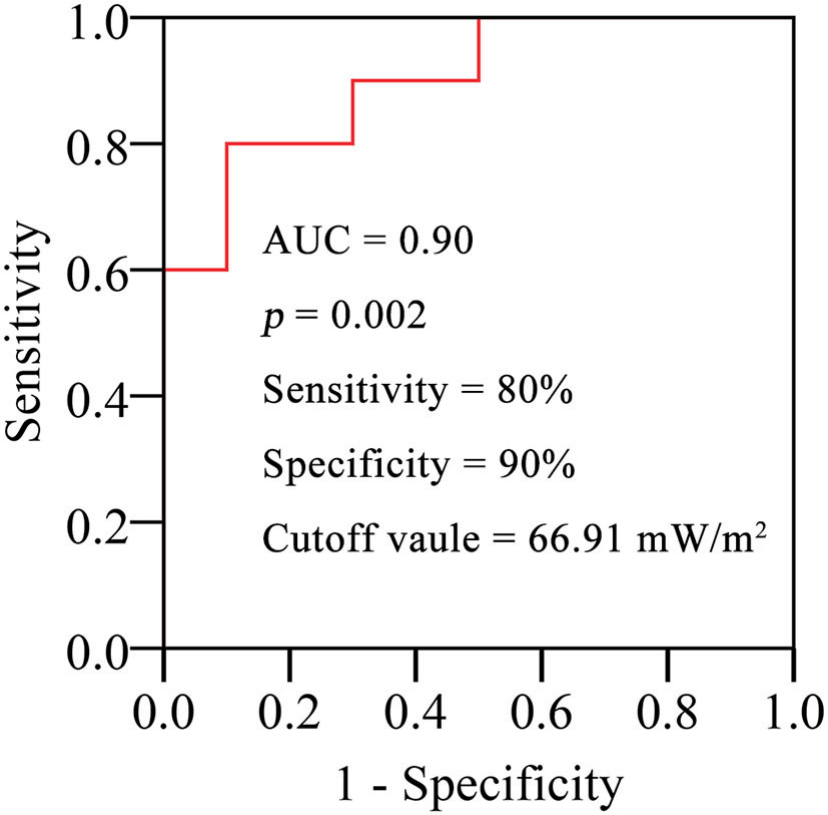
ROC curves analysis of $\overset{.}{E}$ in the pulmonary artery for diagnosing PH-CHD.

ROC curve analysis was performed to evaluate the diagnostic value of $\overset{.}{E}$ in PH-CHD patients (Figure 7). It shows that $\overset{.}{E}$ has a high AUC, sensitivity, and specificity.

## Discussion

PH is one of the most complex and devastating complications of CHD. Various studies (4–6) have demonstrated that the biomechanical mechanism underlying this disease has a major role in PH-CHD development.

Early studies on hemodynamics in PH focused mainly on the metric of WSS, its action on vascular morphology/function, and disease progression. However, several other hemodynamics studies have shown that EL is a key factor in evaluation of hemodynamic disorders (23–25).

Here, we provided new clues to explore more deeply the multiple pathologies of PH-CHD using non-invasive CFD. In the present study, we hypothesized that the hemodynamic parameter of EL could be used to explain the role of abnormal flow dynamics on PH-CHD. To control age-related variation, BSA was used to normalize subject-specific EL ($\overset{.}{E}$).

Based on CFD calculations, the flow-dynamic features were significantly different between PH-CHD patients and control subjects. A high flow rate and velocity [in L/(min·m^2^)] in PA branches were observed only in PH-CHD patients (V_meannorm_: 19.63 ± 6.223 vs. 11.94 ± 3.651, *p* = 0.004; V_maxnorm_: 50.05 ± 13.882 vs. 29.98 ± 8.031, *p* = 0.001) (Figure 3). Expansion of the MPA is one reason for this finding because it causes relative narrowing of the LPA and RPA. This conclusion (Table 2) was supported by a statistical comparison of the two groups with regard to D_MPAnorm_ as well as D_MPA_/D_AO_ and D_MPA_/D_(LPA+RPA)_ ratios. Vortex flow, which occurred during diastole, was observed only in control subjects. The streamlines were relatively steady in PH-CHD patients.

Sanz et al. (10) showed that velocity was a sensitive index for PH evaluation and was closely correlated to pressure and resistance in pulmonary circulation. Tang et al. (4) compared flow patterns between people with and without PH. They found that obvious turbulent flow occurred in a model of a normal PA, a finding that is in accordance with the present study. However, they demonstrated that the flow rate in the PA was higher than in normal subjects: This is exactly the opposite of what we discovered. The difference may have been because of the types of PH explored in these two studies. PH-CHD is a flow-induced disease. Pulmonary blood flow is increased in these patients due to congenital cardiovascular malformations. Abnormal blood flow in pulmonary circulation alters expression of flow-sensitive vascular regulatory factors and is the essential trigger for the development and progression of PH (26–28).

WSS denotes the force generated by the friction between blood flow and the endothelium. It is considered to be a vital biomechanical parameter for evaluation of blood flow–related diseases (29, 30). In healthy people, pulmonary endothelial cells can adapt to a normal range of shear stress. Prolonged abnormal WSS damages the function of pulmonary vessels by destroying endothelial structure and interfering with signal conduction. This process is closely related to PH-CHD development.

WSS has become a “hot topic” in the study of PH-CHD in recent years. Several studies have shown that WSS is a sensitive parameter for evaluating the function of endothelial cells (31, 32). Kheyfets et al. (33) found that WSS was closely related to the elasticity and resistance of the pulmonary artery. Tang et al. (4) explored the WSS of the PA in five PH patients and five control subjects. They showed that the WSS of the MPA was decreased significantly in patients with PH, which was about 20% that of the control group. Figure 4 shows that lower WSS was found in the MPA of PH-CHD patients than in the control subjects. These results are in accordance with those reported previously (4, 9).

In healthy individuals, blood flow is arranged so that EL is low and normal cardiovascular circulation is maintained (34, 35). However, complex cardiovascular malformations in patients with PH-CHD can promote inefficient interactions between pulmonary blood flow and pulmonary structure, which increase EL. The cumulative effect of EL places an extra burden on the heart and may contribute to pulmonary vascular remodeling. Thus, understanding the influence of interrupted flow patterns and altered morphology on EL and the role of increased EL in PH-CHD may benefit clinical diagnosis and treatment.

Nagao et al. (15) explored energy dissipation in healthy people and patients with CTEPH before and after balloon pulmonary angioplasty (BPA) using phase-contrast magnetic resonance imaging. They found that EL was significantly higher in CTEPH patients than in healthy people and that BPA decreased EL. Preoperative EL was an independent and sensitive indicator that predicted patient outcomes.

We showed that EL and $\overset{.}{E}$ were significantly higher in patients with PH-CHD than in non-PH patients (60.41 ± 46.551 vs. 26.34 ± 18.175 mW, *p* = 0.031; 121.60 ± 64.820 vs. 45.15 ± 25.302 mW/m^2^, *p* = 0.007, respectively). In fact, PH-CHD is a right ventricular–pulmonary artery (RV-PA) coupling disease (14). Increased pulmonary arterial pressure and pulmonary arterial resistance gradually damage the structure and function of the right ventricle. EL indicates the compensation of ventricular work. Higher EL indicates that the ventricle must do more work to maintain the stability of the circulatory system. Hence, in patients with PH-CHD, excessively high EL increases the burden on the right ventricle and the risk of right-heart failure. Thus, we provide a theoretical basis for future CFD research of the RV-PA coupling of PH-CHD.

On the other hand, studies have examined EL in patients with CHD extensively. Those studies show that abnormal vessel morphology has a significant effect on EL and the long-term prognoses of patients (36, 37). In the present study, there is a positive relationship between EL and the flow rate of PA but a poor relationship with the morphology of PA (Figure 5). Considering that the development of cardiovascular morphology and function are different in children of different age groups, we use BAS to normalize EL. $\overset{.}{E}$ was positively correlated with the morphology and flow rate in the PA (Figure 6). It implies that age is a strong confounding factor, and normalized EL is a better predictive parameter. Changes in PA morphology, relatively stenosed PA branches, and increased PA flow rate are the main factors of Ė. ROC curve analysis revealed that the Ė was sensitive diagnostic characteristics in PH-CHD (Figure 7). All these results imply that EL is an important factor in PH-CHD evaluation. However, it is worth noting that the cutoff values are just for reference due to the limited cases.

## Limit of the Study

Our study had limitations. Ten PH-CHD patients and 10 age-matched controls were enrolled. Due to the relatively small sample size, only two patients were diagnosed as having irreversible PH-CHD based on postoperative follow-up data. Thus, we cannot offer a clear cutoff for operable and inoperable patients. However, we did identify hemodynamic parameters that were significantly different between the two groups. These results lay a foundation for further study on the reversibility of PH-CHD in a large population of patients. Our simulation assumed that the vessel wall was rigid, and the influence of vascular elasticity on hemodynamics was ignored. However, we aimed to reveal the relationship between hemodynamic indices and PH-CHD by comparing the hemodynamics of the two groups. Therefore, the properties of the vessel wall were simplified in this simulation. Each case was simulated under the same vessel condition, so the rigid-wall hypothesis had little effect on the results.

## Conclusion

$\overset{.}{E}$ is a potential biomechanical parameter for PH-CHD evaluation. The alteration of $\overset{.}{E}$ is closely related to the morphology and flow rate of the PA. This study may offer a new clue for exploring the potential physiopathologic mechanism of PH-CHD and provide more intuitive information for clinicians to make appropriate clinical decisions.

## Data Availability Statement

All datasets generated for this study are included in the article/supplementary material.

## Ethics Statement

This study was approved by the Institutional Health Research Ethics Board of the Shanghai Children's Medical Center, Shanghai Jiao Tong University School of Medicine, and written informed consent was obtained from parent/legal guardian of participants.

## Author Contributions

LW undertook CFD calculations, statistical analyses, and manuscript preparation. JL and ZX contributed to the study design, data collection, and manuscript preparation. YZ and MZ organized the clinical data. JX, JS, and ZT participated in model reconstruction. All authors contributed to the article and approved the submitted version.

## Conflict of Interest

The authors declare that the research was conducted in the absence of any commercial or financial relationships that could be construed as a potential conflict of interest.
